# CD279 mediates the homeostasis and survival of regulatory T cells by enhancing T cell and macrophage interactions

**DOI:** 10.1002/2211-5463.12865

**Published:** 2020-05-13

**Authors:** Yang Liu, Yuting Gao, Huiqin Hao, Tiezheng Hou

**Affiliations:** ^1^ Department of Pathology College of Basic Medical Sciences Shanxi University of Chinese Medicine Jinzhong 030619 China

**Keywords:** immune check point and T cell survival, macrophage, PD‐1 (CD279), PDL‐1 (CD273) and PDL‐2 (CD274), Treg

## Abstract

CD279 is a cell surface protein predominantly expressed on T cells. Its ligands CD273 and CD274 are expressed on antigen‐presenting cells and tumors. CD279 has been shown to act as an important immune check point by inhibiting CD8 T cell activation, and antibodies against CD279 enhance T cell‐mediated cytotoxic function. However, whether CD279 has other functions in CD4 T cell homeostasis or in mediating T cell interactions with antigen‐presenting cells remains unclear. In the present study, we show that antibody‐mediated inhibition of CD279 reduces T cell survival in bone marrow *in vivo*. Unexpectedly, CD279 blockade also compromised regulatory T cell and macrophage interactions by reducing their contact time. The observation that the CD273 antagonist had little effect suggests that CD274 (the second ligand of CD279) plays a more central role in contact between conventional T cells (Tcon) and macrophages. The results of the present study suggest that both CD279 ligands contribute to the interaction length between T cells and macrophages as a mechanism of maintaining Treg homeostasis. Furthermore, CD273 and CD274 are not redundant ligands because CD274 may have unique effects on Tcon in this complex immune axis. Therefore, ligand selection for check point blockade as a tool for cancer immunotherapy has important implications with respect to anti‐tumor T cell activation and the avoidance of side effects.

## Graphical abstract

CD279 is a key immune check point. We show that antibody‐mediated inhibition of CD279 reduces T‐cell survival in bone marrow. CD279 blockade compromised Treg and macrophage interactions by reducing their contact time. The observation that the CD273 antagonist had little effect suggests that CD274 (the second ligand) plays a more central role in contact between Tcon and macrophages.

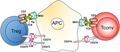

**Abbreviations**
DC: dendritic cell
IL: interleukin
PD‐1: program cell death protein‐1
Tcon: conventional T cells
TCR: T cell receptor
Treg: regulatory T cells

Immune activation is essential for the body to fight against foreign invasion or mutated self‐like tumors. However, the immune system is also tightly controlled. Overwhelmed immunity and autoimmunity are both avoided by immune regulation to prevent self‐damage. CD279, also known as program cell death protein‐1 (PD‐1), is a 55 kDa transmembrane glycoprotein comprising a immunoreceptor tyrosine‐based inhibitory motif that belongs to CD28 superfamily [1, 2]. CD279 is mainly, but not exclusively, expressed on T cells in both CD4 and CD8 compartments [3, 4, 5]. It has basal expression at low level on T cells and is unregulated on activated and exhausted T cells [6, 7, 8]. Unlike CD28, CD279 inhibits T cell stimulation and suppresses the T cell‐mediated immune response [9]. CD279 binds its two ligands CD273 (or PDL‐1) and CD274 (or PDL‐2) [10, 11]. Their expression is restricted to the cells that interact with T cells including antigen‐presenting cells [12] and tissue resident cells [13] in the periphery [14, 15]. The ligation of CD279 with its ligands transmits inhibitory signals to T cells and slows down their effector functions [16, 17, 18, 19, 20]. Therefore, CD279 is a key regulator of immune tolerance of self‐reactive T cells [21] and controls autoimmune diseases [22].

The idea of CD279 as an immune check point has been harnessed by tumor immunotherapists [23]. Blockade of CD279 pathway enhances T cell receptor (TCR) and CD28 downstream signaling and ultimately promotes anti‐tumor immunity [24]. Indeed, pembrolizumab and nivolumab, both of which are anti‐CD279 monoclonal antibodies, have shown promising tumor killing effects and have been approved by the US Food and Drug Administration to treat melanoma and non‐small cell lung cancer clinically. Although ant‐CD279 and anti‐CD273/274 therapy as a novel cancer treatment has become an increasingly profitable area for the pharmaceutical industry to invest in, it is not without any side effects, such as over activating inappropriate immune responses. First, not all cancer patients respond to anti‐CD279 treatment, which indicates that CD279 is not the only immune inhibitory protein. More importantly, targeting CD279 axis nonspecifically activates all responding T cells including those with self‐reactivity [25]. These cells potentially recognize their own body and are responsible for autoimmunity. Indeed, autoimmune diseases such as autoimmune thyroiditis and neuropathy have been found in some patients during trials. This suggests that self‐tolerance is broken in CD279 immune checkpoint blockade. Therefore, understanding the mechanism of anti‐CD279 on immune regulatory functions is important and requires immediate attention.

In the present study, we investigated the function of CD279 on T cell homeostasis and its interactions with antigen‐presenting cells. Using adoptive transfer, we investigated the migration of CD4^+^ T in lymphocyte null recipient mice. At day 2 of reconstitution, donor CD4^+^CD25^+^ regulatory T cells (Treg) were more markedly measurable in host bone marrow compared to the spleen, lymph node and peripheral blood. This suggests that unexpectedly bone marrow provides an unique survival or proliferation niche for Treg. Furthermore, CD279 blockade markedly reduced donor Treg number. Our observation suggests that CD279 plays an unexpected role in Treg homeostatic survival in the resting state. At day 6, Treg and possibly activated T cells are also present in the spleen. In recipient mice receiving anti‐CD279, the percentage of CD4^+^CD25^+^ T cells is significantly greater than that in untreated mice. This suggests that, as predicted, CD279 blockade induced T cell activation. Although CD279 as an immune check point is evident, the mechanism of CD279 with respect to promoting Treg survival and the functional differences between its two ligands on macrophages remain incompletely characterized. To address these questions, we also examined the interaction between T cells and macrophages in an *in vitro* co‐culture model. For the first time, we demonstrated that antagonizing both CD279 ligands reduced the Treg contact time with macrophages. This suggests that CD279 plays a role in promoting Treg interactions with macrophages. In brief, CD279 is an important receptor for resting Treg homeostasis. After activation, CD279 on Treg and possibly conventional T cells (Tcon) inhibits their stimulation. Our work suggests that CD279 has different kinetic roles in subsets of T cells. Although CD273 and CD274 showed synergetic effects in Treg and macrophage interactions, CD274, but not CD273, contributed to Tcon and macrophage contact duration. This result suggests that the two ligands have different effects on Treg and Tcon.

## Materials and methods 

### Mice and tissue harvest

All experiments were performed in accordance with local laws and with the approval of University Ethics Committee of Shanxi University of Traditional Chinese Medicine. C57BL/6 and Rag2^−/−^ mice were all bred and maintained in the animal house. Tissues were harvested and meshed through a nylon net. For cells originating from bone marrow and spleen, erythroid cells were lysed by Lympholyte‐M (ACL5031; Fisher Scientific, Waltham, MA, USA) and cells were washed using fluorescence‐activated cell sorting buffer at 350 ***g*** for 5 min.

### Magnetic cell sorting

CD4^+^ T cells were enriched by negative selection using a MagniSort kit (8804‐6821‐74; Invitrogen, Carlsbad, CA, USA) in accordance with the manufacturer’s instructions. Briefly, cells were labeled with biotinylated anti‐CD8, CD11b, CD19, CD24, B220, CD49b, Ly‐6G and γδ TCR and secondary streptavidin‐coated magnetic beads. After placing the cells in a magnetic field, untouched CD4^+^ T cells are free in solution. CD25^+^ Treg were sorted by negative selection (11463D; Invitrogen) and the positive fraction comprised CD25^−^ Tcon. Macrophages were enriched by magnetic separation of F4/80^+^ cells by positive selection using a MagniSort kit (8802‐6863‐74; Invitrogen).

### Adoptive transfer and migration assay

CD4^+^ T cells from spleen of C57BL/6 mice were purified as described above. CD4^+^ T cells were pre‐treated with anti‐CD279 (clone RMP1‐14) (BE0146; Bio X Cell, West Lebanon, NH, USA) or Rat IgG2a isotype control (Clone 2A3) (BE0089; Bio X Cell) and injected (i.v.) into Rag2^−/−^ mice. Tissues including peripheral blood, bone marrow, spleen and mesenteric lymph nodes were harvested at days 2 and 6.

### Flow cytometry

Cells were counted and Fc receptors were blocked using anti‐CD16/CD32 (clone 2.4G2) (catalog no. 553141) from Becton‐Dickinson (Franklin Lakes, NJ, USA) to prevent non‐specific binding. Cells for immunofluorescence staining were labelled with monoclonal antibodies: anti‐CD3e FITC (clone 145‐2C11) (catalog no. 11‐0031‐86) or Armenian hamster IgG isotype control FITC (clone eBio299Arm) (catalog no. 11‐4888‐81), anti‐CD11c APC (clone N418) (catalog no. 17‐0114‐82) or Armenian hamster IgG isotype control APC (clone eBio299Arm) (catalog no. 17‐4888‐82), and anti‐B220 PE (clone HIS24) (catalog no. 12‐0460‐82) or mouse IgG2b kappa isotype control PE (clone eBMG2b) (catalog no. 12‐4732‐82) from eBioscience (Carlsbad, CA, USA) and anti‐CD4 PerCP (clone RM4‐5) (catalog no. 553052) and rat IgG2a, κ isotype control PerCP (clone R35‐95) (catalog no. 553933) from BD Pharmingen (San Diego, CA, USA) at 4 °C for 30 min. Excessive antibodies were washed off and labeled cells were analyzed using a flow cytometer (BD ACCURI C6). Data were analyzed usinf flowjo (Tree Star Inc., Ashland, OR, USA) and prism (GraphPad Software Inc., San Diego, CA, USA).

### Interaction assay and imaging analysis

To distinguish Tcon or Treg from macrophages, Tcon or Treg was labeled with Cell Trace Far Red (FR) (C34564; Thermo Fisher). Treg, Tcon and macrophages were resuspended at 1 × 10^6^/mL. Cells were mixed into 100 µL in the presence of rat IgG2a isotype control (PA5‐33214; Invitrogen), anti‐CD273 (clone TY25) (14‐5986‐85; eBioscience) or anti‐CD274 (clone MIH5) (14‐5982‐82; eBioscience). Culture suspension was loaded into eight‐well Lab‐Tek class chamber slides (C7182l Sigma, St Louis, MO, USA). Interaction events were recorded as video after 5 min to allow cells to settle. Video recordings was made at 5 s per frame for 240 frames (total time = 20 m). Cell contact and interactions were detected and recorded using fluorescent microscope scanning system (LSM 410; Carl Zeiss, Oberkochen, Germany). Video and images were analyzed using volocity 4 (Quorum Technologies Ltd, Lewis, UK) and imagej (NIH, Bethesda, MD, USA). respectively. All culture was carried out in RPMI 1640 with 10% fetal bovine serm in a moisturized incubator with 5% CO_2_ at 37 °C.

### Statistical analysis

Student’s *t*‐test was used for the statistical analysis. *P* < 0.05 was considered statistically significanct.

## Results

### CD279 is essential for Treg homeostasis in bone marrow

**Fig. 1:**
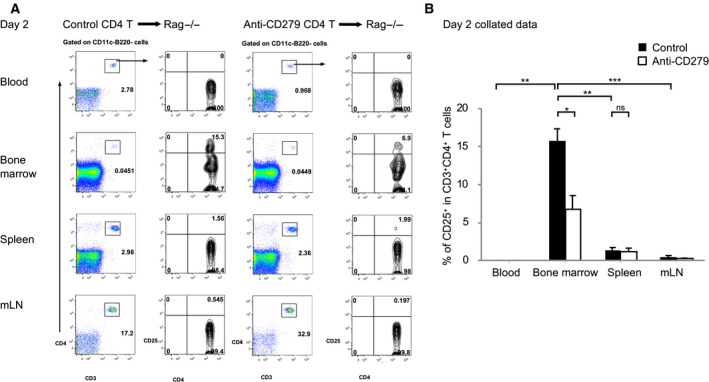
CD279 enhances resting Treg survival in the bone marrow. (A) Donor CD4^+^CD25^+^ T cells were adoptive transferred by i.v. injection into a Rag^−/−^ recipient with a lack of lymphocytes. Wild‐type CD4^+^ T cells were treated in the presence or absence of blocking CD279 antibody. Blood, bone marrow, spleen and mesenteric lymph node were harvested at day 2 after transfer and cells were stained by flow cytometry. CD11c^+^ DC and B220^+^ B cells were gated out and the percentages of CD3^+^CD4^+^ T cells and CD25^+^ T cells are shown. The data are representative of five independent experiments with a minimum of three mice per group. (B) Collated data from (A) are shown with the mean ± SD (error bars). Percentages of CD25 are plotted with isotype control (closed) and anti‐CD279 treated (open) conditions. Student’s *t*‐test was performed and *P* < 0.05 was considered statistically significant (ns, not significant; *P < 0.05, **P < 0.01, ***P < 0.001).

Treg has basal level of CD279 expression in the resting state. However, whether CD279 has a role in Treg homeostasis *in vivo* is unknown. To test this, we adoptive transferred CD4^+^ T cells from wide type donor spleen into lymphocyte deficient Rag^−/−^ mice. Donor T cells were detectable in blood, bone marrow, spleen and mesenteric lymph nodes at day 2 after transfer. However, CD4^+^CD25^+^ T cells were significantly enriched in host bone marrow than elsewhere (Fig. 1A). This indicates that either Treg preferentially migrates to bone marrow or bone marrow provides specific survival niche for Treg, but not Tcon. More importantly, CD279 inhibition using blocking antibody led to a reduced number of donor Treg by 50% (Fig. 1A,B). This suggests that CD279 plays a role in Treg survival or turnover during homeostasis.

### CD279 inhibits lymphopenic induced proliferation of T cell

**Fig. 2:**
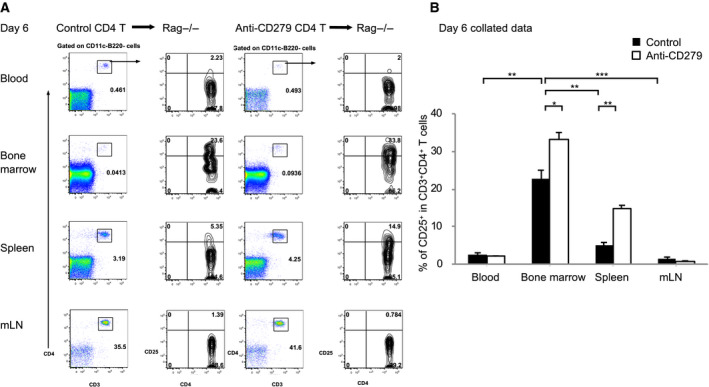
CD279 inhibits the proliferation of activated T cells. The experiment was set‐up as shown in Fig. 1, although tissues were harvested at day 6 after transfer. (A) Donor CD4^+^CD25^+^ T cells were adoptive transferred by i.v*.* injection into Rag^−/−^ recipient with a lack of lymphocytes. Wild‐type CD4^+^ T cells were treated in the presence or absence of blocking CD279 antibody. Cells from blood, bone marrow, spleen and mesenteric lymph node were stained by flow cytometry. CD11c^+^ DC and B220^+^ B cells were gated out and percentages of CD3^+^CD4^+^ T cells and CD25^+^ T cells are shown. The data are representative of five independent experiments with a minimum of three mice per group. (B) Collated data from (A) are shown with the mean ± SD (error bars). Percentages of CD25 are plotted with isotype control (closed) and anti‐CD279 treated (open) conditions. Student’s *t*‐test was performed and and *P* < 0.05 was considered statistically significant (*P < 0.05, **P < 0.01, ***P < 0.001).

Six days after transplant, donor T cells expanded markedly in the recipient, particularly in the lymph node. This demonstrates that the effect of lymphopenic induced proliferation took place at this stage of adoptive transfer. CD4^+^CD25^+^ T cells appeared in the spleen and blood but not the lymph node (Fig. 2A). This indicates the activation of T cells and upregulation of CD25 on the transfer of CD4^+^ T cells. Furthermore, in mice receiving anti‐CD279 treated cells, CD25^+^ T cells increased by 30% and 100% in the bone marrow and spleen, respectively, compared to those in untreated mice (Fig. 2A,B). This suggests that CD279 blockade induced T cell proliferation. This is consistent with the notion that CD279 negatively regulates T cells by inhibiting his activation. Overall, these data provide further evidence that CD279 has multiple functions in different T cell subsets and kinetic roles during the immune response.

### CD273 and CD274 play a role in the contact between Treg and macrophages

**Fig. 3:**
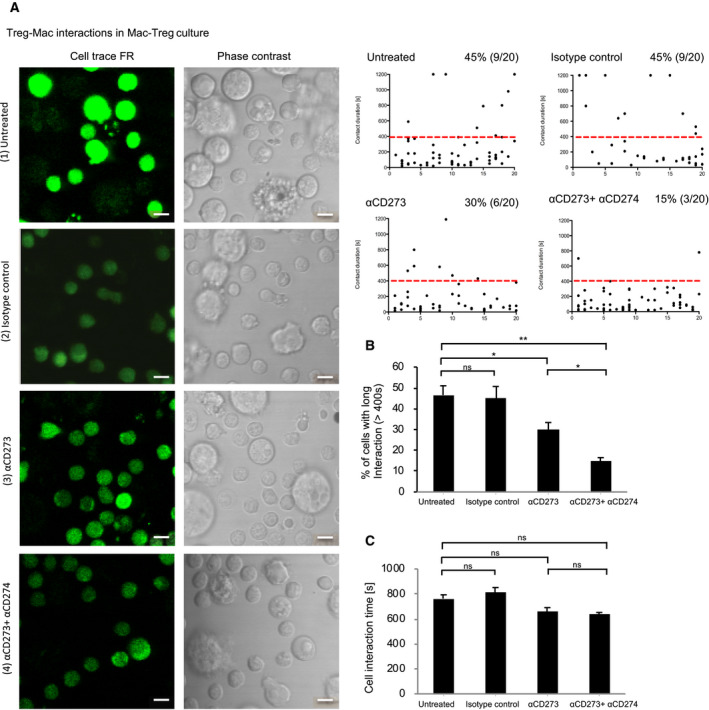
CD273 and CD274 enhance contact between Treg and macrophages. (A) Purified Treg and macrophages were co‐cultured in the absence (1) or presence of isotype control (2), anti‐CD273 (3), or anti‐CD273 and anti‐CD274 antibodies (4). Macrophages are generally bigger in size under a microscope than Treg. Treg are fluorescence labeled with Cell Trace FR shown in pseudo green to distinguish from macrophages. Each black dot represents one interaction from a randomly chosen Treg and each cell may encounter one or multiple interactions. The contact time between Treg and macrophages was analyzed using video recordings with fluorescence and phase contrast microscopy. Contact time above 400 s above the dotted red line was regarded as a meaningful interaction and graphed against total contact time as a percentage. Scale bars = 10 µm. The data shown are representative of six independent experiments. (B) Collated data of percentage of cells with long interactions (> 400 s) are shown with the mean ± SD (error bars). (C) Collated data of the contact time from (A) with the mean ± SD (error bars). Student’s *t*‐test was performed and and *P* < 0.05 was considered statistically significant (ns, not significant; *P < 0.05, **P < 0.01).

To investigate the mechanism of CD279 supporting Treg survival, we investigated the interaction between Treg and macrophages in a co‐culture assay. We randomly chose individual Cell Trace FR labeled Treg and measured their contact time with macrophages. We found that 45% of cells had long interactions (> 400 s) with macrophages in untreated or isotype control antibody treated conditions. Blocking CD273 (ligand of CD279) reduced this to 33% of cells with a long contact time. Antagonizing both CD279 ligands (CD273 and CD274) reduced the long contact of Treg with macrophages by 66% (Fig. 3A,B). This suggests that either ligand plays a role in promoting Treg interactions with macrophages, potentially via its receptor CD279. This is consistent with the notion that CD279 supports Treg homeostatic survival. Interestingly, ligand blockade did not compromise the average contact time of long interactions (Fig. 3C). This suggests that CD279 may contribute to the commitment of long interactions but, once committed, CD279 had no effect on the duration of the interactions.

### CD274 but not CD273 contributes to Tcon interactions with macrophages

**Fig. 4:**
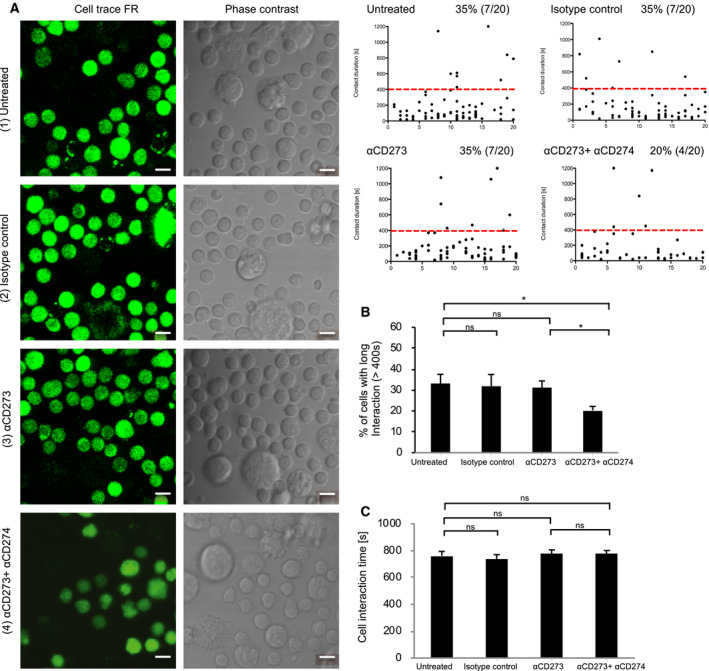
CD274 enhances contact between Tcon and macrophages. (A) Purified Tcon and macrophages were co‐cultured in the absence (1) or presence of isotype control (2), anti‐CD273 (3) or anti‐CD273 and anti‐CD274 antibodies (4). Macrophages are bigger in size under microscope than Tcon. Tcon are fluorescence labeled with Cell Trace FR shown in pseudo green to distinguish from macrophages. Each black dot represents one interaction from a randomly chosen Tcon and each cell may encounter one or multiple interactions. The contact time between Tcon and macrophages was analyzed using video recordings with fluorescence and phase contrast microscopy. The percentage of cells with long interactions (> 400 s) and the total contact time with long interactions is shown. Scale bars = 10 µm. The data are representative of six independent experiments. (B) Collated data of the percentage of cells with long interactions (> 400 s) are shown with the mean ± SD (error bars). (C) Collated data of contact time from (A) are shown with the mean ± SD (error bars). Student’s *t*‐test was performed and and *P* < 0.05 was considered statistically significant (ns, not significant; *P < 0.05).

To test whether CD279 ligand also contributes to the contact between Tcon and macrophages, we also measured the contact time between Tcon and macrophages under ligand blockade conditions. Interestingly, the contact time between Tcon and macrophages is more than 20% less than that of Treg and macrophages. Inhibition of CD273 alone had no effect and blocking both CD273 and CD274 reduced long contact by 30% (Fig. 4A,B). These data suggest that CD279 contributes less to cell interactions with macrophage than Treg did. Similar to Treg, CD274 did not affect the contact time once Tcon was committed to long interactions (Fig. 4C).

## Discussion

CD279 antagonizes the TCR signaling pathway by a different mechanism compared to that of CD152 despite their synergy in the negative regulation of T cell activation [26]. Apart from differential expression pattern between CD279 and CD152 and their ligands in various cell subpopulations and tissues, they differ in their downstream signaling pathways with respect to inhibiting the upregulation of glucose metabolism and Akt phosphorylation mediated by CD3/CD28. In CD152 suppression, okadaic acid and PP2A and removal of CD80/CD86 play a vital role, whereas CD279 signaling prevents phosphoinositide 3‐kinase activation via the immunoreceptor tyrosine‐based switch domain in its cytoplasmic tail [27]. These actions lead to distinct changes in the T cell transcriptional profiles and, ultimately, effector functions at different stages of immunity. CD152 is considered to control T‐cell proliferation early in an immune response, mostly in lymph nodes. By contrast, CD279 regulates T cells later after activation, primarily in the periphery [28].

CD279 is a critical regulator in Treg homeostasis and, as a result, the CD279 pathway supports Treg‐mediated immunotolerance. CD279^−/−^ Treg rapidly phosphorylated Stat5 and divided before upregulation of proapoptotic marker Fas and downregulation of survival marker Bcl‐2. Therefore, increased CD279 expression on central‐memory Treg after interleukin (IL)‐2 induced Treg proliferation rescued Treg from being overly activated and exhausted [29]. T cells interacting with dendritic cells (DCs) *in vitro* has been reported previously [30]. Treg has longer contact with DC than Tcon does. However, whether this is also true for other type of antigen‐presenting cells has not been investigated previously. In the present study, we developed a co‐culture system to study the interactions between Treg and macrophages by investigating whether CD279 plays any part in the process. The ligation of CD279 receptor with its ligands CD273 or CD274 has been shown to drive a negative signal to T cells [18, 19]; therefore, it can inhibit T cell activation and effector function, including IL‐4 and IL‐21 [31]. Blocking CD279 breaks the tolerance and results in the priming of T cells; therefore, the interaction between T cells and resting DCs is crucial for maintaining tolerance via the CD279 pathway [32]. However, whether this ligation is also connected to Treg homeostasis remains unknown.

Structurally, CD279 is engaged differently by its two ligands based on changes in its backbone nuclear magnetic resonance signals. CD274 has 34‐fold greater binding affinity for CD279 than CD273 as a result of its smaller dissociation rate [2]. Indeed, CD273 and CD274 have been shown to have opposing functions in airway inflammation and hyper‐reactivity [33]. Our finding of a function for CD274 in macrophages is consistent with the observation of the inhibition of T cells by alternatively activated macrophages via Stat6‐dependent expression of CD274 [34]. Taken together, this suggests that ablating CD274 and CD279 ligation could be a tool in therapeutic intervention.

In conclusion, the present study has confirmed a role for CD279 in the maintenance of Treg homeostasis. This is closely associated with CD279‐CD273/CD274 ligation and interaction between T cells and macrophages. Disrupting this their contact might inhibit CD279 and Treg function. The other novelty of our findings is that CD274, the second ligand, has a unique role in Tcon and macrophages compared to the minimal role of CD273. Overall, our work has further clarified the functional differences between CD279 ligands and suggests that this could be important with respect to ligand blocking strategies in the treatment of tumor patients.

## Conflict of interest

The authors declare no conflict of interest.

## Author contributions

HH and TH conceived and designed the project. YL acquired the data. YL and YG analysed and interpreted the data. HH and TH wrote the paper.
